# Polyethyleneimine Modified Expanded Vermiculite-Supported Nano Zero-Valent Iron for Cr(VI) Removal from Aqueous Solution

**DOI:** 10.3390/ma18132930

**Published:** 2025-06-20

**Authors:** Xinyu Yang, Yan Mu, Lina Zhang, Dan Sun, Tiantian Jian, Weiliang Tian

**Affiliations:** 1 Engineering Laboratory of Chemical Resources Utilization in South Xinjiang of Xinjiang Production and Construction Corps, College of Chemistry and Chemical Engineering, Tarim University, Alar 843300, China; xyyang_1995@163.com (X.Y.); 17835236190@163.com (Y.M.); 15619076231@163.com (L.Z.); dan@taru.edu.cn (D.S.); 2 Instrumental Analysis Center of Tarim University, Alar 843300, China; jtt18168224512025@163.com

**Keywords:** vermiculite, nZVI, adsorption, reduction

## Abstract

In order to develop an efficient, environmentally friendly heavy metal ions adsorbent, the amino-modified expanded vermiculite-supported nano zero-valent iron (nZVI@PEI/EVMT) was prepared by using polyethyleneimine (PEI) as the functional reagent and expanded vermiculite (EVMT) as the carrier. The characterization results of nZVI@PEI/EVMT confirm that the PEI modification did not destroy the crystal configuration of EVMT, and when nano zero-valent iron (nZVI) was successfully loaded onto the PEI/EVMT surface, the value of saturation magnetic field was 41.5 emu/g, which could be separated from solution with magnet. The performance of Cr(VI) adsorption onto nZVI@PEI/EVMT was studied, showing that the ideal mass ratio for nZVI@PEI/EVMT was 1:1, and the removal capacity was largest when solution pH was 2. After four adsorption–desorption cycles, the adsorption amounts remained 40.1 mg/g. The Cr(VI) adsorption onto nZVI@PEI/EVMT was more consistent with a pseudo-second-order kinetics equation. Isotherm adsorption data accord with the Langmuir model, which suggests that the adsorption was the monolayer, the maximum adsorption amount was 116.2 mg/g at 30 °C and pH 2, and the adsorption was spontaneous and endothermic. It was inferred that the adsorption mechanisms included electrostatic attraction, reduction, chemical complexation, and co-precipitation.

## 1. Introduction

The heavy metal chromium (Cr) was one of the most toxic chemicals to the environment [1], which ranged from +2 to +6 in oxidation states; among them, Cr(VI) ions had the characteristics of high toxicity, persistence, and hard degradation [2,3]. Cr(VI) commonly existed in wastewater such as in electroplating, leather, and dyestuff, easily entered into the environment, which presented a significant risk to ecosystems [4,5]. The adsorption was a popular cleanup technique for heavy metal pollution because of its good availability, ease of use, and high efficiency [6,7]. In response to the increasingly serious heavy metal pollution, the development of an environmentally friendly and sustainable heavy metal adsorbent was necessary.

Nano zero-valent iron (nZVI) was commonly employed for treatment of Cr(VI)-polluted water [8,9,10], but the direct application of nZVI might cause the rapid loss of nZVI. Due to its small particle size, nZVI exhibited a strong tendency to agglomerate into larger particles, therefore reducing the reactivity of nZVI [11,12,13]. To overcome these limits, it was found that using carbon materials, clay minerals, and polymer as carriers to load nZVI would better solve the agglomeration problem [14,15]. Shi et al. [16] investigated the Cr(VI) removal by bentonite-loaded nZVI (B-nZVI), which showed that the distribution of nZVI on porous bentonite could effectively inhibit the agglomeration of nZVI particles, and the Cr(VI) removal efficiency by B-nZVI was higher than that of bare nZVI, significantly improving the reactivity. Vermiculite (VMT) was a kind of nonmetallic mineral with layered structure, loading nZVI onto VMT would bolster the dispersion of nZVI, then, enhancing the Cr(VI) adsorption efficiency. Nevertheless, the bare raw VMT carrier for heavy metal ions adsorption was poor, and could be chemically expanded to crack the interlayer lattice and widen the interlayer spacing to obtain the expanded vermiculite (EVMT), and the adsorption capacity of the EVMT was larger than the raw VMT. However, the EVMT was of single surface-active groups [17], and the adsorption efficiency could be improved by adding functional groups into the interlayer of the EVMT [18]. Polyethyleneimine (PEI) was the linear macro molecule, composed of numerous active amine groups, which could be used for the adsorption of diverse species [19]. PEI exhibited excellent complex ability for Cr(VI) ions [20,21], but PEI was easily dissolved in aqueous solution, which could not be used as adsorbent alone and was often used as an amino modification reagent for adsorbents. Nie et al. [22] prepared the new adsorptive membrane via combining polyacrylonitrile (PAN) with crosslinked PEI, which showed great Cr(VI) removal capacity, the maximum adsorption amount being 89.16 mg/g. Thus, for increasing the capability of Cr(VI) adsorption, the EVMT was modified with PEI solution, and PEI was grafted into the interlayer of the EVMT to prepare PEI-modified EVMT composites (PEI/EVMT); it served as the carrier for loading nZVI particles, and the use of PEI-modified EVMT supported nZVI composite (nZVI@PEI/EVMT) for Cr(VI) removal was explored in this paper.

The aim of this study was to synthesize nZVI@PEI/EVMT, characterization, and explore mechanisms for Cr(VI) adsorption, the specific synthesis procedure included (1) incorporation of PEI into EVMT, and (2) reduction in Fe^0^ via the liquid-phase reduction method. The effects of pH, temperature, and initial concentrations for Cr(VI) removal were investigated, the mechanisms were explored by kinetics analysis and material characterization, the prepared nZVI@PEI/EVMT adsorbent showed good adsorption capacity and reusability, and the synthesis route for nZVI onto PEI/EVMT had a foremost role in the expansion of iron-based functional materials. Furthermore, this research provided a fundamental knowledge of Cr(VI) removal, and allows us a common understanding for remediation of Cr(VI)-polluted water.

## 2. Materials and Methods

### 2.1. Reagent

The raw vermiculite was obtained from Xinjiang Yuli Xinlong Vermiculite Co., Ltd. (Yuli, China); iron chloride hexahydrate (FeCl_3_·6H_2_O), sodium borohydride (NaBH_4_), hydrogen peroxide (H_2_O_2_), hydrochloric acid (HCl), and glutaraldehyde were purchased from Sinopharm Group Chemical Reagent Co. Ltd. (Shanghai, China). Polyethyleneimine (PEI, molecular weight of 25,000), potassium dichromate (K_2_Cr_2_O_7_), and sodium hydroxide (NaOH) were bought from Aladdin Reagent Co., Ltd. (Shanghai, China). Deionized water was purified via bubbling N_2_.

### 2.2. Preparation of nZVI@PEI/EVMT

#### 2.2.1. Synthesis of PEI Modified EVMT (PEI/EVMT)

The raw vermiculite was soaked in H_2_O_2_ (15 wt%) solution and chemically expanded for 48 h to obtain EVMT, it was then crushed to fit through a 200-mesh screen for later use. The synthesis method of PEI-modified EVMT (PEI/EVMT) was conducted according to an earlier study and optimized for our experimental conditions [23]. A total of 1.0 g EVMT was dispersed into 50 mL PEI methanol solution (5 wt%) and continuously stirred for 2 h; then, 50 mL glutaraldehyde solution (2 wt%) was added for a cross-linking reaction for 10 h at 30 °C, and the finished products were cleaned with distilled water and vacuum-dried for 8 h at 60 °C to obtain PEI-modified EVMT composites (PEI/EVMT).

#### 2.2.2. Synthesis of nZVI@PEI/EVMT

The material was synthesized in a 250 mL three-neck flask, and 0.5 g PEI/EVMT and 2.41 g FeCl_3_·6H_2_O were dispersed into 100 mL deionized water by mechanically stirring. Then, under nitrogen protection, NaBH_4_ solution (0.50 g in 50 mL deionized water) was added into the flask drop by drop; after that, the chemical reaction continued for 40 min, and finally, the products were separated with a magnet and washed with deionized water twice, then vacuum-dried at 50 °C to obtain nZVI@PEI/EVMT.

### 2.3. Characterization Techniques

The morphology of samples was determined using a scanning electron microscope (SEM, ZEISS Sigma 300, Oberkochen, Germany). An X-ray diffractometer (XRD, Rigaku SmartLab SE, Tokyo, Japan) was used to measure the crystal diffraction of the samples. The surface functional groups of EVMT, PEI/EVMT, and nZVI@PEI/EVMT were analyzed using a Fourier transform infrared spectrometer (FT-IR, Thermo Scientific Nicolet iS50, Waltham, MA, USA). The hysteresis loop of the adsorbent was measured by using a vibrating sample magnetometer (LakeShore7404, Westerville, OH, USA). A nitrogen adsorption–desorption curve (Micromeritics ASAP 2460, Norcross, GA, USA) was employed to calculate the specific surface area of nZVI@PEI/EVMT. The elemental species of the sorbent were determined with a X-ray photoelectron spectrometer (XPS, Thermo Scientific ESCALAB 250Xi, Waltham, MA, USA).

### 2.4. Adsorption Process

The batch adsorption was under the baseline conditions of a Cr(VI) initial concentration of 60 mg/L, nZVI@PEI/EVMT dosage of 1 g/L, 30 °C, solution of pH 2, and contact time of 180 min. Briefly, A conical flask containing 100 mL Cr(VI) solution was filled with 0.1 g adsorbent and adsorbed for 180 min, and the experiments were conducted three times. Using the 1,5-diphenylcarbohydrazide method, an ultraviolet-visible spectrophotometer (U-3000, Shimadzu, Kyoto, Japan) set at 540 nm was used to measure the Cr(VI) concentration. Flame atomic absorbance spectrometer (AA-6300C, Shimadzu, Kyoto, Japan) was used to detect the total Cr concentration.

### 2.5. Kinetics Experiments

Pseudo-first-order (Equation (1)) and pseudo-second-order kinetics models (Equation (2)) were used to fit experimental data [24].

(1) $$\ln(q_{e}-q_{t})=\ln q_{e}-k_{1}t$$

(2) $$t/q_{t}=1/(k_{2}q_{e}^{2})+t/q_{e}$$

where *q*_e_ (mg/g) and *q*_t_ (mg/g) were the adsorption amount; *k*_1_ (min^−1^) and *k*_2_ (g/mg min) were kinetics constants, respectively.

### 2.6. Adsorption Isotherm Experiments

Under the baseline conditions, the initial Cr(VI) concentration was adjusted from 60 to 400 mg/L. Langmuir (Equation (3)) and Freundlich models (Equation (4)) were utilized to analyze the obtained data [25]:

(3) $$q_{e}=q_{\max}K_{L}C_{e}/(1+K_{L}C_{e})$$

(4) $$q_{e}=K_{F}{C_{e}}^{1/n}$$

where *q*_e_ was defined as above, *C*_e_ (mg/L) represented the equilibrium concentration and *q*_max_ (mg/g) represented the saturation adsorption amount. Langmuir constant *K*_L_ represented the affinity of adsorbent, the Freundlich constant *K*_F_ represented the adsorption ability, and *n* was the equilibrium constant.

The entropy change (Δ*S*^0^), enthalpy change (Δ*H*^0^), and Gibbs free energy change (Δ*G*^0^) could be calculated by the following formulas (Equations (5)–(7)), and the intercept of the ln*K*_d_ versus 1/T curve could be used to calculate Δ*H*^0^ and Δ*S*^0^, respectively [26].

(5) $$K_{d}=q_{e}/C_{e}$$

(6) $$\Delta G^{0}=-RT\ln K_{d}$$

(7) $$\ln K_{d}=-\Delta H^{0}/(RT)+\Delta S^{0}/R$$

## 3. Results and Discussion

### 3.1. Characterization of Materials

**Figure 1:**
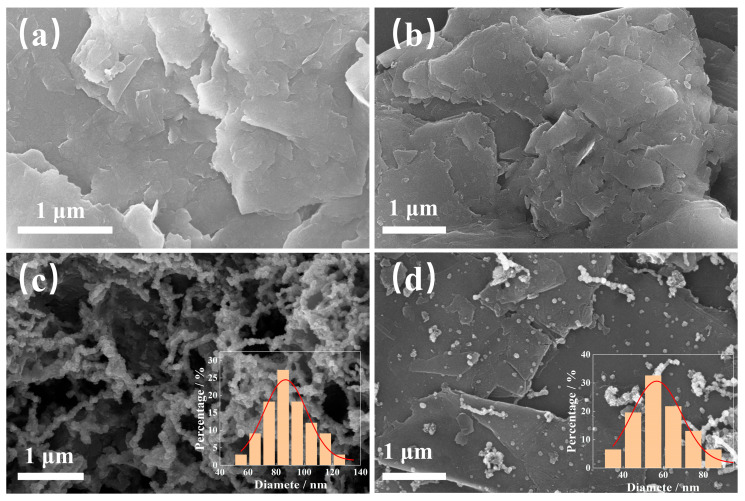
SEM images of (**a**) EVMT; (**b**) PEI/EVMT; (**c**) nZVI; and (**d**) nZVI@PEI/EVMT.

Figure 1 shows the images of EVMT, PEI/EVMT, nZVI, and nZVI@PEI/EVMT, respectively. As it is presented in Figure 1a that the EVMT showed a layered structure with a smooth surface, and the edge of the layered structure was flat and curved shape, after PEI modification, the SEM image of PEI/EVMT (Figure 1b) was more fragmented compared with the EVMT, and the layered structure was still visible, showing that the PEI modification did not destroy the crystal configuration of the EVMT. Figure 1c shows that nZVI had irregular spherical particles, which clustered together, showing obvious agglomeration. Figure 1d shows that the spherical particles were distributed onto PEI/EVMT, revealing that the Fe^0^ was loaded on a PEI/EVMT carrier, although a small number of nZVI still agglomerated together, but the agglomeration phenomenon had been significantly improved compared with bare nZVI. The insets in Figure 1c,d are the particle size distribution of nZVI, which showed that the average size of bare nZVI was approximately 87.1 nm, and the average size of nZVI in nZVI@PEI/EVMT composite was approximately 56.8 nm, suggesting that loading nZVI onto a PEI/EVMT carrier could reduce the size of nZVI particles.

**Figure 2:**
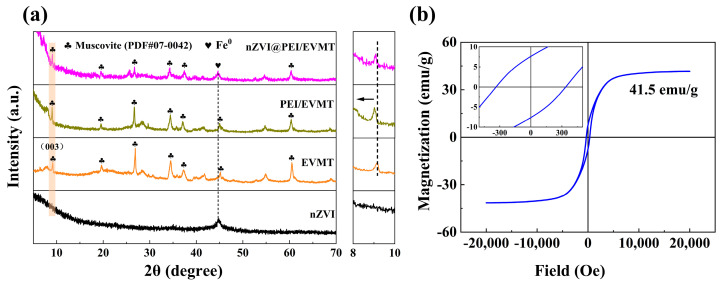
(**a**) XRD patterns of samples; (**b**) magnetization curves of nZVI@PEI/EVMT.

The XRD patterns of nZVI, EVMT, PEI/EVMT, and nZVI@PEI/EVMT are shown in Figure 2a, the nZVI presented strong diffraction peak at 44.7°, which corresponded to the 110 crystal diffraction of Fe^0^ [27,28], the EVMT was mainly contained muscovite phase (JCPDS 07–0042) [29], the similar peaks could be also observed in PEI/EVMT and nZVI@PEI/EVMT patterns, suggesting that the layered structure of EVMT was not destroyed, which was preserved during the modification [30], and it was consistent with the SEM results. Furthermore, the observed peak at 9.1° corresponded to the (003) crystal plane of EVMT, which shifted towards the smaller degree in the PEI/EVMT and nZVI@PEI/EVMT pattern, revealing that the PEI molecules had been grafted into the interlayer of the EVMT, resulting in larger interlayer spacing for the PEI/EVMT [31,32]. nZVI@PEI/EVMT showed a characteristic peak of Fe^0^ at 44.7°, suggesting that the nZVI particles were successfully loaded onto PEI/EVMT; additionally, there were no characteristic peaks of iron oxides in nZVI and nZVI@PEI/EVMT patterns, showing that little Fe^0^ was oxidized into amorphous iron oxides [33].

Figure 2b showed the saturation magnetization curve of nZVI@PEI/EVMT. The saturation magnetization value was 41.5 emu/g, which was lower than bare nZVI (143 emu/g) from previous research [34]. This might be attributed to the addition of the PEI/EVMT carrier. The ferromagnetic characteristics of nZVI@PEI/EVMT were confirmed based on the coercivity value of 337.2 Oe, which could be separated from solution using a magnet.

**Figure 3:**
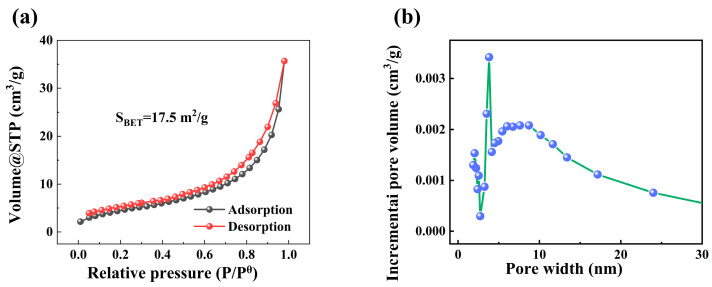
(**a**) N_2_ adsorption–desorption isotherm; (**b**) pore diameter distribution curve.

The N_2_ adsorption–desorption isotherm of nZVI@PEI/EVMT is displayed in Figure 3a, where it was shown that the curve was not closed, possibly because there were many adsorption sites on the nZVI@PEI/EVMT surface. At low relative pressures, some adsorption sites might be saturated, while others remained available, which could continue to adsorb molecules during desorption, therefore leading to a non-closure for the curve [35]. The BET surface area of nZVI@PEI/EVMT was 17.5 m^2^/g, which was larger than the bare nZVI (8.82 m^2^/g) in previous research [36]. It was more advantageous for adsorption. From the pore diameter distribution curve in Figure 3b, the nZVI@PEI/EVMT is found to be mainly composed of mesopores.

**Figure 4:**
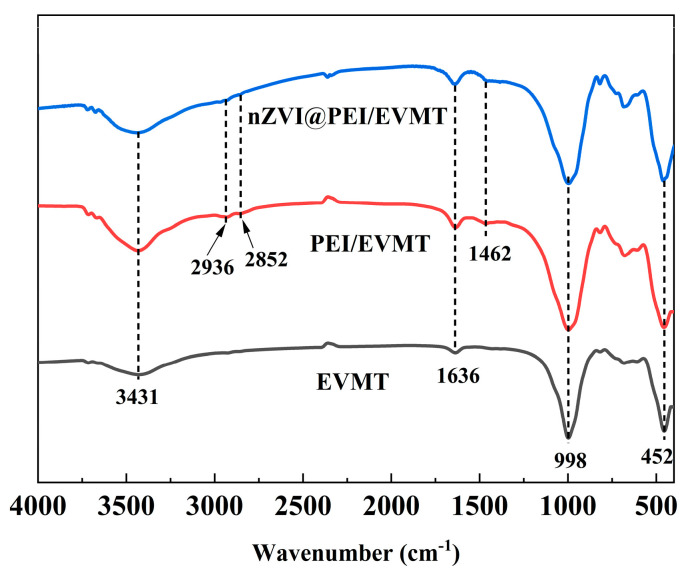
FT-IR spectra of EVMT, PEI/EVMT, and nZVI@PEI/EVMT.

The FT-IR spectra of EVMT, PEI/EVMT, and nZVI@PEI/EVMT are shown in Figure 4. For EVMT, the characteristic peak at 998 cm^−1^ corresponded to a stretching vibration of Si–O–Si bonds, while a peak at 452 cm^−1^ was the bending vibration of Si–O bonds. The peaks at 3431 and 1636 cm^−1^ were attributed to the stretching vibration of interlayer water molecules and O–H bending vibration, and the region between 600 and 800 cm^−1^ was associated with metal–oxygen bond vibrations (e.g., Al–O and Mg–O). The PEI/EVMT spectrum exhibited new vibrational peaks at 2936 and 2852 cm^−1^, assigning to C–H stretching vibration in the polyethyleneimine molecules. Additionally, a new peak observed at 1462 cm^−1^ corresponds to the C–N bond of PEI. Combined with XRD analysis results of the enlarged interlayer spacing in PEI/EVMT, these observations confirmed the polyethyleneimine was successfully intercalated into the interlayer of EVMT. For nZVI@PEI/EVMT, the enhancement of the 452 cm^−1^ peak could be explained by the overlapping of the Fe–O vibration with the original Si–O bending vibration, suggesting the successful loading of nZVI onto the EVMT carriers.

### 3.2. Effect of nZVI@PEI/EVMT Mass Ratio

**Figure 5:**
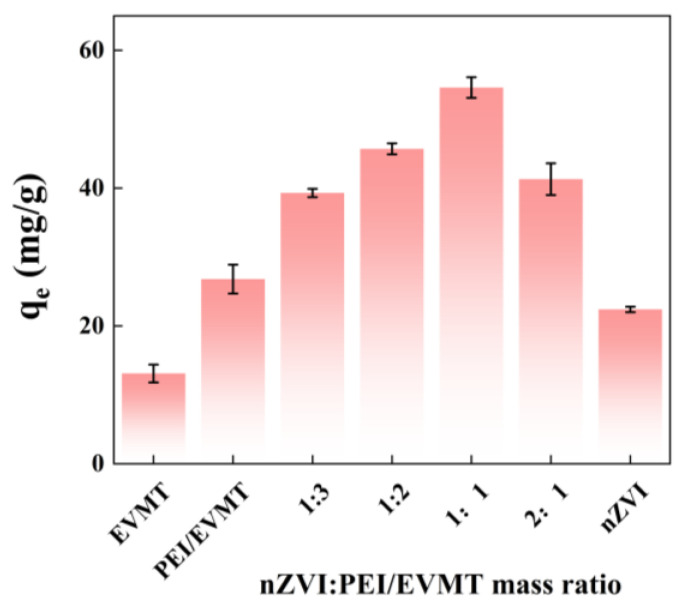
Effect of nZVI@PEI/EVMT mass ratio.

The effect of the nZVI@PEI/EVMT mass ratio is illustrated in Figure 5. The bare EVMT, PEI/EVMT, and nZVI showed that Cr(VI) removal capacity was 13.1, 26.8, and 22.4 mg/g, respectively. The Cr(VI) adsorption onto EVMT only depended on physical adsorption, thus, the removal efficiency was poor. When EVMT was modified by PEI, the PEI/EVMT showed higher adsorption capacity than EVMT, and because the modified amino active groups could react with Cr(VI) ions to improve the adsorption capacity, and the adsorption capacity of nZVI@PEI/EVMT was strongly affected by nZVI and PEI/EVMT mass ratio, when nZVI and PEI/EVMT mass ratio was 1:3, the adsorption amount was 39.3 mg/g, and as it increased to 1:2 and 1:1, the adsorption amount was improved to 45.7 and 54.6 mg/g; the increasing adsorption amount might be because of the fact that with more nZVI particles loading on PEI/EVMT, more reactive sites could enhance the removal performance. However, when nZVI and PEI/EVMT mass ratio were increased to 2:1, the excessive nZVI particles on PEI/EVMT led to the aggregation problem, and the Cr(VI) removal capacity decreased. The optimal mass ratio of nZVI@PEI/EVMT was 1:1.

### 3.3. The Effect of Initial pH on Adsorption

**Figure 6:**
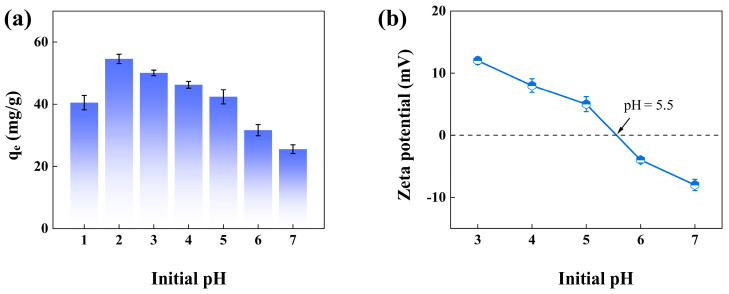
(**a**) Effect of pH; (**b**) zeta potential of nZVI@PEI/EVMT.

The solution pH value was adjusted using 0.1 mol/L HCl and NaOH, respectively. As seen in Figure 6a, the adsorption performance of nZVI@PEI/EVMT was more favorable under acidic conditions, and adsorption capacity was largest when pH was 2. pH value could affect the existing forms of Cr(VI) ions. HCrO_4_^−^ ions were typically predominant species between pH 2 and 6, and when pH increased over 6, the Cr(VI) species changed to CrO_4_^2−^ [37]. Figure 6b shows the potential of nZVI@PEI/EVMT under different pH values, where the zero-point charge value of nZVI@PEI/EVMT was 5.5, suggesting that when solution pH is lower than 5.5, the active amino groups in nZVI@PEI/EVMT protonated with free H^+^ in solution; therefore, nZVI@PEI/EVMT was positively charged, while Cr(VI) existed mainly as negatively charged HCrO_4_^−^ in acidic medium, the positively charged adsorbent could generate electrostatic attraction to adsorb HCrO_4_^−^, and as the solution pH was larger than 5.5, nZVI@PEI/EVMT was negatively charged and electrostatic repulsion occurred between adsorbent and HCrO_4_^−^, CrO_4_^2−^, and Cr_2_O_7_^2−^ ions, causing the decline in uptake capacity. Additionally, the adsorption capacity decreased at pH = 1, since Cr(VI) species changed to neutral H_2_CrO_4_ molecules at pH = 1 [38,39,40], indirectly decreasing the number of negatively charged Cr(VI) ions, and the electrostatic attraction of adsorbent to Cr(VI) ions became weakened, and therefore, the next experiments were conducted under pH 2.

### 3.4. Adsorption Kinetics

**Figure 7:**
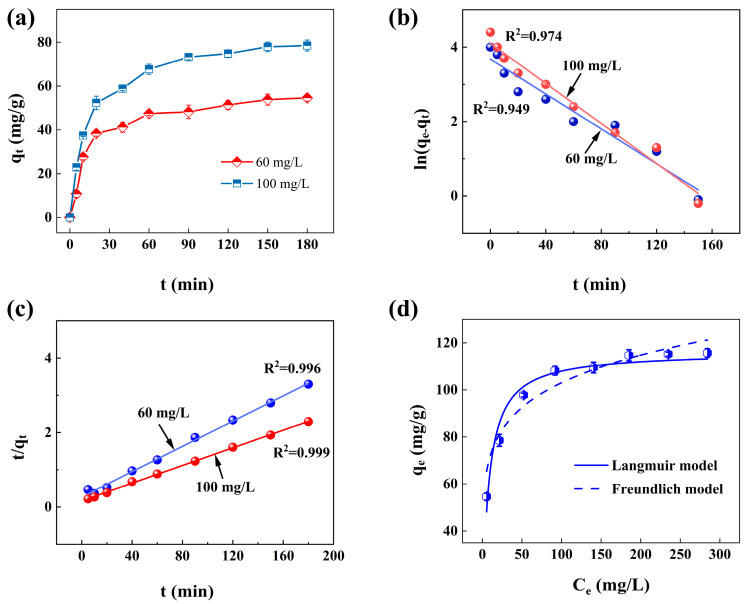
(**a**) Effect of contact time; (**b**) pseudo-first-order kinetics fitting plots; (**c**) pseudo-second-order kinetics fitting plots; and (**d**) equilibrium isotherm for adsorption.

Figure 7a presents the effect of contact time at the initial concentration of 60 and 100 mg/L, which revealed that the adsorption rate was significantly fast firstly and slowly reached an equilibrium; furthermore, when the initial concentration was 100 mg/L, the adsorption capacity of nZVI@PEI/EVMT was larger than that of 60 mg/L, and the effective adsorption could be assigned to the unique interaction between Cr(VI) and nZVI@PEI/EVMT. To evaluate mechanisms of Cr(VI) removal, pseudo-first-order and pseudo-second-order kinetics equations were used to fit the data.

The kinetics fitting results are presented in Figure 7b,c, the calculated kinetics constants are in Table 1, and correlation coefficients (R^2^) of the pseudo-second-order kinetics model were larger than pseudo-first-order kinetics model, revealing that the pseudo-second-order kinetics equation fit perfectly with experimental data. The pseudo-second-order kinetics equation was used according to the assumption that chemical adsorption was the speed control step for the whole adsorption process [41], and it could be considered that the mechanisms of nZVI@PEI/EVMT for Cr(VI) adsorption were mainly affected by reduction, chemical complexation, and co-precipitation, and additionally, the equilibrium adsorption capacity calculated from the pseudo-second-order kinetics equation (*q*_e,cal_) fit better with the experimental results (*q*_e,exp_).

### 3.5. Adsorption Isotherms

The influence of initial concentrations were studied to illustrate how nZVI@PEI/EVMT reacted with Cr(VI). The isotherm models were used to fit the data, which could be seen in Figure 7d. The obtained parameters were in Table 2. Obviously, the Cr(VI) removal by nZVI@PEI/EVMT was better fit by the Langmuir model (R^2^ = 0.958) due to the higher correlation coefficient, suggesting that the adsorption was a monolayer adsorption rather than multilayer and heterogeneous adsorption [42]. The maximum adsorption amount was 116.2 mg/g at 30 °C, *K*_F_ value was 50.2, and 1/n was 0.156 (0.1 < 1/n < 2), which indicated the easy separation of Cr(VI) from liquid phase [43].

### 3.6. Adsorption Thermodynamics

**Figure 8:**
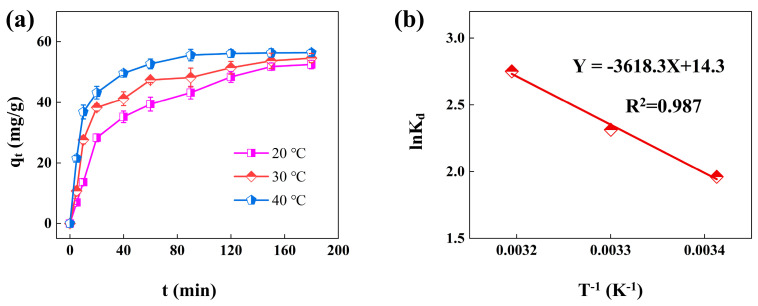
(**a**) Adsorption capacity for Cr(VI) removal by nZVI@PEI/EVMT under different temperatures; (**b**) the linear dependence of lnK_d_ on 1/T.

Figure 8a shows the Cr(VI) adsorption onto nZVI@PEI/EVMT with different adsorption temperatures (20, 30, and 40 °C) and that the adsorption capacity gradually increased as the adsorption time proceeded, and it should be noted that the increasing temperature improved the adsorption rate and adsorption capacity, which indicated that the higher temperature was beneficial for adsorption process. The thermodynamic parameters of Δ*S*^0^ and Δ*H*^0^ could be calculated via the ln*K*_d_ versus 1/T curve (Figure 8b). The results are shown in Table 3, the Δ*G*^0^ were all less than zero, showing the adsorption could be reacted spontaneously, and the Δ*S*^0^ was 118.9 J/mol K, which showed the adsorption was a process of increasing entropy, and the Δ*H*^0^ was 30.1 kJ/mol and was endothermic [44,45], hence, the increasing of temperature was conducive to Cr(VI) adsorption.

### 3.7. Materials Reusability

**Figure 9:**
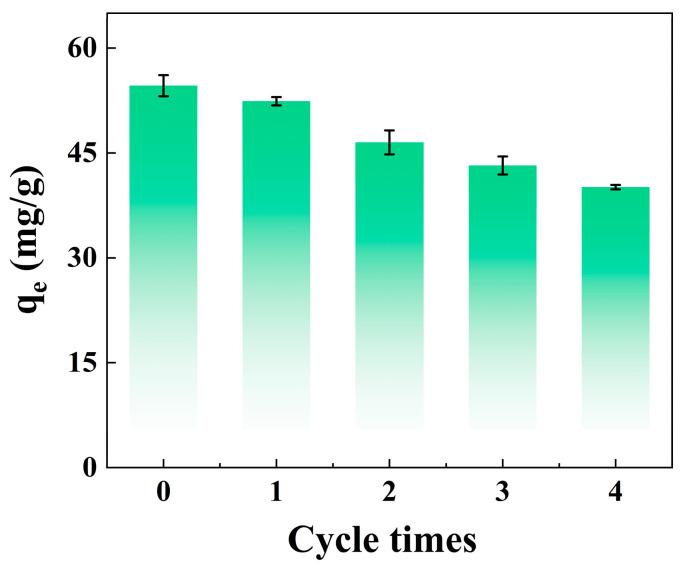
Reusability evaluation of nZVI@PEI/EVMT.

The nZVI@PEI/EVMT was collected by centrifuge, and 0.1 mol/L HCl was used for desorption of nZVI@PEI/EVMT, and the recovered nZVI@PEI/EVMT could be renewable via 0.5 mol/L NaBH_4_, then, the same volume and initial concentration of Cr(VI) solution was added for the cyclic experiment. Figure 9 reveals that the corresponding adsorption capacity of nZVI@PEI/EVMT was reused four times. With the increasing recycling times, the adsorption capacity decreased slowly, which might be because the nZVI particles were consumed gradually during the adsorption; additionally, partial Cr(VI) ions adsorbed in nZVI@PEI/EVMT were not eluted and still occupied the adsorption sites. After four adsorption–desorption cycles, the adsorption capacity decreased from 54.6 mg/g to 40.1 mg/g, with less than 5 mg/g decreasing in average per cycle, and the results show that the nZVI@PEI/EVMT was an economical adsorbent with good reusability [46].

## 4. Adsorption Mechanisms

**Figure 10:**
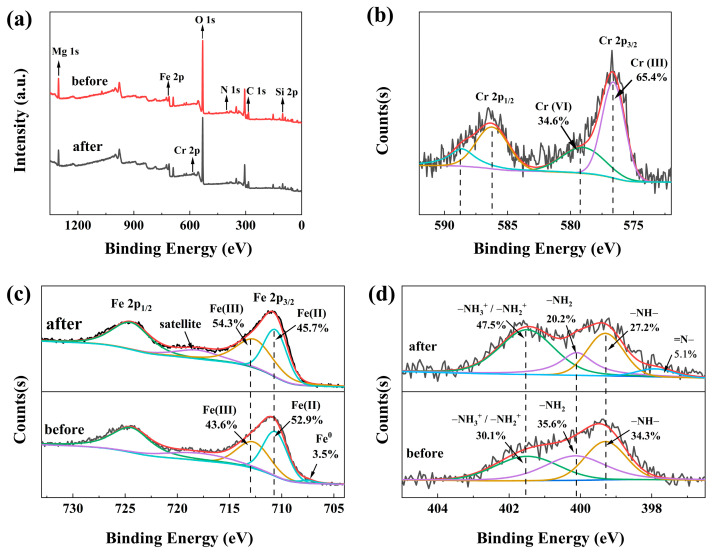
(**a**) XPS wide survey of nZVI@PEI/EVMT; high-resolution spectra of (**b**) Cr 2p; (**c**) Fe 2p; and (**d**) N 1s.

To further analyze the mechanisms, XPS was used to determine the elemental composition and morphological changes. As seen from the Figure 10a, the main elements of nZVI@PEI/EVMT were Mg, Fe, O, C, Si, and N, where the N element was from the modified reagent of PEI, which confirmed that the N-containing groups were successfully grafted onto EVMT; after adsorption, the signal of Cr 2p appeared in nZVI@PEI/EVMT, confirming the successful uptake of Cr(VI) onto nZVI@PEI/EVMT. Furthermore, the spectrum of Cr 2p (Figure 10b) suggested that peaks at 588.8 and 579.2 eV belonged to Cr(VI), while peaks at 586.2 and 576.7 eV corresponded to Cr(III) [47]. This revealed that Cr(VI) ions were reduced to Cr(III) during adsorption. According to the characteristic peak areas, the Cr element was mainly existed in Cr(III) species on nZVI@PEI/EVMT.

In Fe 2p spectrum (Figure 10c) shows the peak at 707.3 eV belonged to Fe^0^ before adsorption [48], suggesting the nZVI was successfully loaded onto PEI/EVMT, which is consistent with XRD analysis results. However, the Fe^0^ peak disappeared after adsorption, which suggested that Fe^0^ involved a mechanism for Cr(VI) reduction. Moreover, peaks at 712.9 and 710.7 eV were assigned to Fe(III) and Fe(II), respectively [49], indicating that nZVI@PEI/EVMT was partially oxidized during the preparation process, and peak areas of Fe(III) and Fe(II) changed after adsorption because the redox potential of HCrO_4_^−^/Cr^3+^ (+1.35 V) was larger than Fe^2+^/Fe^0^ (−0.44 V) and Fe^3+^/Fe^2+^ (+0.77 V) [50]; thus, HCrO_4_^−^ could be spontaneously reduced by Fe^0^ and Fe^2+^ (Equations (8) and (9)), and additionally, the generated Cr(III) and Fe(III) ions were adsorbed on nZVI@PEI/EVMT, which could have reacted to produce Fe(III)/Cr(III) co-precipitates (Equations (10) and (11)) [51].

**Figure 11:**
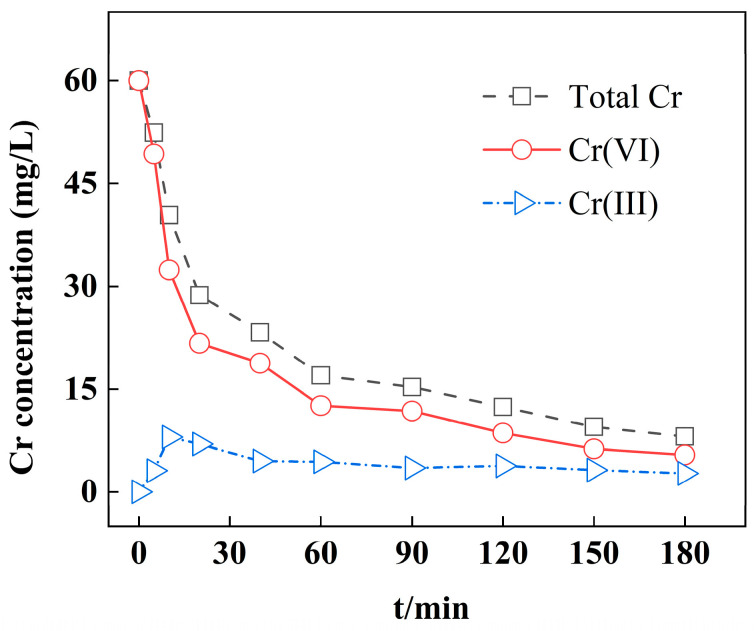
The change in Cr species during adsorption.

In the N 1s spectrum (Figure 10d), the peaks at 401.5, 400.1, and 399.3 eV were attributed to -NH_3_^+^/-NH_2_^+^, -NH_2_, and-NH-, respectively [52]. Importantly, the peak area of -NH_3_^+^/-NH_2_^+^ increased from 30.1% to 47.5% after adsorption, indicating that the amine groups were easily protonated during adsorption, and the protonated active groups could adsorb negatively charged Cr(VI) ions by electrostatic attraction, which was advantageous for adsorption. In addition, the new characteristic peak of =NH- (5.1%) appeared at 397.5 eV after adsorption, suggesting that the amine groups from the PEI molecules could act as the electron donor to change Cr(VI) into Cr(III) (Equations (12) and (13)). It is noteworthy that the peak areas of -NH_2_ and -NH- both decreased after adsorption, which might because that the generated Cr(III) ions could react with these groups via chemical complexation, leading to the decreasing of -NH_2_ and -NH- groups. The change in Cr species during adsorption are displayed in Figure 11. It can be seen that the Cr(III) ions were detected at the beginning, and the Cr(III) concentration gradually decreased as the adsorption time proceeded, and this result is consistent with the above analysis, the generated Cr(III) could be co-precipitated by reacting with the Fe(III) ions, and Cr(III) ions could be also complexed with the amine groups to be adsorbed on nZVI@PEI/EVMT, therefore leading to the decrease in Cr(III) concentration.

**Figure 12:**
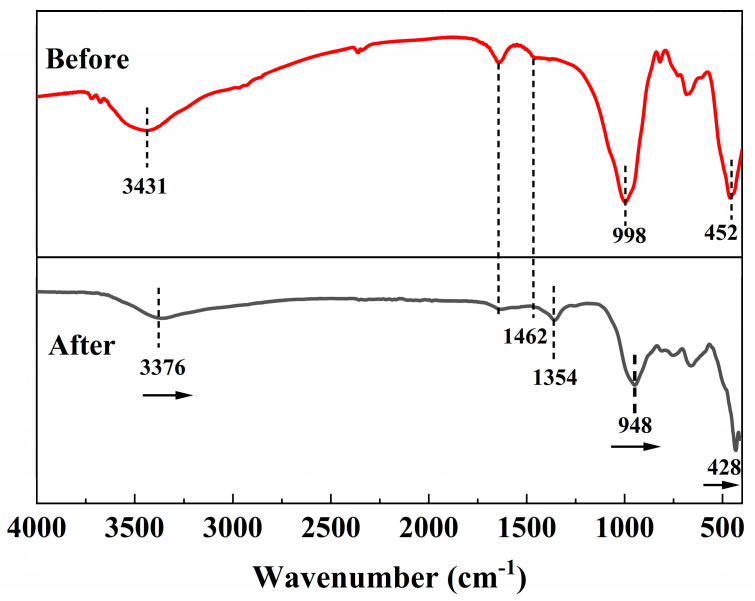
FT-IR spectra of nZVI@PEI/EVMT before and after adsorption.

The FT-IR spectra of nZVI@PEI/EVMT before and after adsorption are shown in Figure 12, the characteristic peaks of nZVI@PEI/EVMT did not change significantly after adsorption, indicating that the nZVI@PEI/EVMT adsorbent was stable, and the crystalline structure of EVMT was better preserved. However, the characteristic peaks at 452, 998, and 3431 cm^−1^ were all shifted to lower wavelength after adsorption, probably because the Cr(VI) anion reacted with the protonated amino group (-NH_3_^+^) from the EVMT interlayer, which weakened the Si-O bond and the metal–oxygen bonds in EVMT [53]. The insertion of Cr(VI) ions changed the interlayer environment of EVMT, and the binding of Cr(VI) with -NH_3_^+^ led to the disappearance of C-N bond at 1462 cm^−1^. Furthermore, a new peak appeared at 1354 cm^−1^ after adsorption, which was attributed to the vibration of the Cr-O bond [54], suggesting that Cr(VI) ions were adsorbed onto nZVI@PEI/EVMT.

(8) $${3\mathrm{Fe}}^{0}+{{2\mathrm{HCrO}}_{4}}^{-}+{14H}^{+}\to {2\mathrm{Cr}}^{3+}+{3\mathrm{Fe}}^{2+}+{8H}_{2}O$$

(9) $${3\mathrm{Fe}}^{2+}+{{\mathrm{HCrO}}_{4}}^{-}+{7H}^{+}\to {\mathrm{Cr}}^{3+}+{3\mathrm{Fe}}^{3+}+{4H}_{2}O$$

(10) $${\mathrm{xCr}}^{3+}+(1-x){\mathrm{Fe}}^{3+}+{3H}_{2}O\to {\mathrm{Cr}}_{x}{\mathrm{Fe}}_{(1-x)}{\left(\mathrm{OH}\right)}_{3}↓+{3H}^{+}$$

(11) $${\mathrm{xCr}}^{3+}+(1-x){\mathrm{Fe}}^{3+}+{2H}_{2}O\to {\mathrm{Cr}}_{x}{\mathrm{Fe}}_{(1-x)}\mathrm{OOH}↓+{3H}^{+}$$

(12) $${{\mathrm{HCrO}}_{4}}^{-}+{7H}^{+}+{3e}^{-}\to {\mathrm{Cr}}^{3+}+{4H}_{2}O$$

(13) $${\mathrm{Cr}}_{2}{O_{7}}^{2-}+14H^{+}+6e^{-}\to 2{\mathrm{Cr}}^{3+}+{7H}_{2}O$$

**Figure 13:**
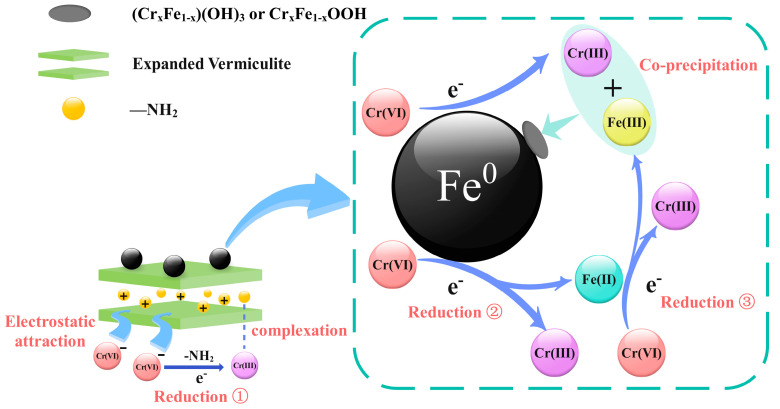
Probable mechanism for Cr(VI) removal.

The above results suggest the mechanisms of Cr(VI) adsorption onto nZVI@PEI/EVMT probably included electrostatic attraction, reduction, chemical complexation, and co-precipitation. Figure 13 shows the adsorption process, the Cr(VI) ions in the solution were rapidly adsorbed onto nZVI@PEI/EVMT by electrostatic attraction, and the adsorbed Cr(VI) could be reduced to Cr (III) by Fe^0^, Fe(II), and amino groups, and the generated Cr(III) and Fe(III) ions could form Fe(III)/Cr(III) co-precipitates; furthermore, the generated Cr(III) ions could also react with the amine groups via chemical complexation. As a result, the Cr(VI) ions could be removed completely.

## 5. Conclusions

A new magnetic adsorbent of nZVI@PEI/EVMT for Cr(VI) elimination was prepared, the nZVI particles were distributed onto a PEI/EVMT carrier, and the agglomeration phenomenon had been significantly improved compared with bare nZVI. The removal capacity was strongly affected by nZVI and PEI/EVMT mass ratio, the best mass ratio of nZVI@PEI/EVMT was 1:1, the Cr(VI) uptake onto nZVI@PEI/EVMT possessed an obvious pH dependence, and the adsorption was more favorable under acidic conditions, with the largest Cr(VI) adsorption capacity at pH 2. The adsorption results could be well described by a pseudo-second-order kinetics model and Langmuir adsorption isotherm. The maximum removal amount was 116.2 mg/g at 30 °C and pH 2. The obtained thermodynamics constants suggested that the adsorption was spontaneous and endothermic. After four instances of adsorption–desorption experiments, the adsorption capacity decreased less than 5 mg/g in average per cycle, showing that nZVI@PEI/EVMT not only had good adsorption capacity, but also was an economical adsorbent, and the mechanism of Cr(VI) adsorption onto nZVI@PEI/EVMT was proved to include electrostatic attraction, reduction, chemical complexation, and co-precipitation.

## Figures and Tables

**Table 1:** Kinetic parameters for adsorption.

C_0_ (mg/L)	*q*_e,exp_ (mg/g)	Pseudo-First-Order			Pseudo-Second-Order		
		*k*_1_ (min^−1^)	*q*_e,cal_ (mg/g)	*R* ^2^	*k*_2_ (g/mg min)	*q*_e,cal_ (mg/g)	*R* ^2^
60	54.6	2.36 × 10^−2^	39.2	0.949	1.05 × 10^−3^	59.2	0.996
100	78.5	2.69 × 10^−2^	59.7	0.974	8.57 × 10^−4^	84.0	0.999

**Table 2:** The Cr(VI) adsorption isotherm constants.

Langmuir			Freundlich		
*K*_L_ (L/mg)	*q*_m_ (mg/g)	*R* ^2^	*K*_F_	*1*/*n*	*R* ^2^
0.131	116.2	0.958	50.2	0.156	0.918

**Table 3:** The Cr(VI) adsorption thermodynamic constants.

Δ*G*^0^ (kJ/mol)			Δ*H*^0^ (kJ/mol)	Δ*S*^0^ (J/mol K)
20 °C	30 °C	40 °C	30.1	118.9
−4.78	−5.83	−7.16		

## Data Availability

The original contributions presented in this study are included in the article. Further inquiries can be directed to the corresponding author.
